# Entodermoscopy: A Useful Tool for the Diagnosis of Cutaneous Larva Migrans

**DOI:** 10.5826/dpc.1102a14

**Published:** 2021-03-08

**Authors:** Claudio Conforti, Arianna Dri, Ludovica Toffoli, Enrico Zelin, Iris Zalaudek, Nicola Di Meo

**Affiliations:** 1 Dermatology Clinic, Hospital Maggiore, University of Trieste, Italy

**Keywords:** entodermoscopy, dermoscopy, larva migrans, tropical disease

## Introduction

Cutaneous larva migrans (CLM) is a zoonotic skin infestation caused by different species of helminths of the hookworm family, clinically characterized by a cutaneous erythematous serpiginous track. It is endemic in tropical countries; however, cases in Western Europe are increasing and usually affect returning travelers. CLM is typically acquired through contact with feces of infected animals, especially when walking barefoot on contaminated soil. Feet are the typical site of infestation, since the parasite penetrates through the skin digging a burrow. The diagnosis of CLM is usually based on morphology of skin lesions. Herein we report 2 cases in which dermoscopy helped the interpretation of cutaneous signs, confirming the clinical diagnosis of CLM.

## Case Presentation

**Figure 1:**
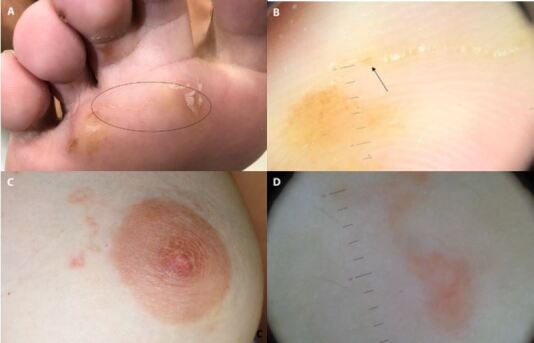
(A) Clinical presentation of cutaneous larva migrans (CLM) of the foot. It presents as a serpiginous white scaly lesion (circle), over slightly erythematous skin. (B) The main dermoscopic features of CLM of the foot are small brown dots (arrow), corresponding to the body of the helminth, and white segmental scales outlining the parasite’s burrow within the skin (DermLite; ×10). (C) CLM of the nipple presenting as a creeping erythematous eruption. (D) Dermoscopy of CLM of the nipple shows a reddish serpiginous track and yellow areas on the background (DermLite; ×10).

The first case concerns a 26-year-old man who came in for consultation for a creeping eruption located on the sole of the left foot. He was a homeless, of South Asian origin, and had immigrated in Italy 2 months prior. He described walking barefoot during the journey. The eruption consisted of a winding lesion of about 5 cm, over slightly erythematous skin (Figure 1A). The patient complained of an itch that had been persisting for many weeks. Dermoscopy (DermLite; ×10) revealed a well-defined yellowish serpiginous course with whitish scales extending across the metatarsal plantar surface of the foot. It represented the path of the parasite within the skin. A small cluster of brown dots was visible at one end of the track, that probably was the correlate of the body of the parasite (Figure 1B). These findings pointed towards the diagnosis of CLM.

A second case, an otherwise healthy 34-year-old woman, presented with an erythematous and pruritic eruption beside the mammary areola (Figure 1C). No other body areas were involved. The disorder developed 1 week after returning from a trip to the Caribbean, during which time she was topless and came into contact with sand. Dermoscopic evaluation showed a reddish serpiginous track and yellow areas on the background (Figure 1D). Integration of clinical and anamnestic data led to a diagnosis of CLM. The body of the parasite was not visible, likely because it was digging the burrow at deeper layers of the skin. Alternatively, it could have been masked by the intense phlogistic reaction of the delicate breast area.

Few reports concerning dermoscopic presentation of CLM are available in literature so far. The body of the helminth has been described as composed of translucent yellow-brownish structureless oval spots in a segmental arrangement, sometimes associated to red dotted vessels in the empty burrow [1,2].

In clinical practice scabies could also be included in the differential diagnosis of an itchy eruption. Upon dermoscopy its lesions are characterized by a typical dark brown triangle, corresponding to the anterior pigmented part of the mite, in contiguity with a whitish curved line representing the burrow. The global appearance is addressed as the jet with contrail sign. Moreover, scabies usually involves multiple peculiar body locations and often affects cohabiting partners.

Both our patients were treated with albendazole 400 mg orally for 3 days. Betamethasone plus fusidic acid ointment was prescribed 2 times per day, in order to reduce local skin inflammation. Favorable clinical response was obtained within 2 weeks.

## Conclusions

The diagnosis of CLM infestation is often based on anamnesis and clinical presentation since it is characterized by the distinctive pruritic serpiginous track involving a single body area. Nevertheless, dermoscopy can be a useful tool when uncertain, as it allows a clearer view of the curvilinear burrow and occasionally the direct visualization of the parasite’s body. Skin infestations are becoming increasingly common due to climate change towards milder temperatures, the frequent trips to tropical countries, and the migration phenomenon, so dermoscopy helps clinicians deal with these emergent pathologies.
